# Metabolic-Modulating Effects of Radiation: Undetectable Yet Deadly—A Review on Radiotherapy

**DOI:** 10.3390/cancers17010054

**Published:** 2024-12-27

**Authors:** Francesco Fiorica, Umberto Tebano, Giuseppe Napoli, Antonella Franceschetto, Marco Muraro, Carlotta Giorgi, Paolo Pinton

**Affiliations:** 1 Department of Clinical Oncology, Section of Radiation Oncology and Nuclear Medicine, AULSS 9 Scaligera, 37122 Verona, Italy; umberto.tebano@auilss9.veneto.it (U.T.); giuseppe.napoli@aulss9.veneto.it (G.N.); antonella.franceschetto@aulss9.veneto.it (A.F.); marco.muraro@aulss9.veneto.it (M.M.); 2 Department of Morphology, Surgery and Experimental Medicine, Section of Pathology, Oncology and Experimental Biology, Laboratory for Technologies of Advanced Therapies (LTTA), University of Ferrara, 48033 Ferrara, Italy; carlotta.giorgi@unife.it (C.G.); paoilo.pinton@unife.it (P.P.)

**Keywords:** Metabolic cancer pathway and radiotherapy, alternative radiation cell killing, radiation synergism

## Abstract

Radiotherapy has traditionally been viewed as a localised treatment modality, primarily inducing DNA damage to eradicate cancer cells. However, emerging research reveals that radiation also profoundly affects cellular metabolism, particularly in reprogramming tumour cells’ energy production and survival pathways. This article explores the role of metabolic reprogramming in the response of cancer cells to radiotherapy, highlighting how these biological processes can be leveraged to enhance treatment efficacy and overcome resistance. Radiotherapy is also discussed, focusing on how metabolic and molecular profiling of tumours can help personalise radiation treatments to improve outcomes.

## Simple Summary

This review explores how radiotherapy disrupts critical metabolic pathways in cancer cells. It highlights vulnerabilities in glucose metabolism, lipid synthesis, and amino acid pathways, which radiotherapy exploits to enhance tumour control. Advancements such as nanoparticle-enhanced and FLASH radiotherapy are discussed for their ability to improve precision and reduce damage to healthy tissues. Tools like FDG-PET scans are emphasised for detecting metabolic changes and guiding therapy combinations, such as precision radiotherapy with metabolic inhibitors. This review also outlines future prospects, including personalised metabolic imaging, integration with systemic therapies, targeting tumour-specific metabolic dependencies, and innovations like immune modulation and ferroptosis induction. These approaches aim to transform radiotherapy from a localised treatment to a systemic strategy that targets cancer’s metabolic complexities, improving both efficacy and patient outcomes.

## 1. Introduction

From a cancer-centric perspective, radiotherapy has been primarily viewed as a localised treatment modality, targeting cancer tissues with ionising radiation to induce DNA damage and cell death [1].

Radiobiology states that radiation affects cancer cells by generating double-strand breaks (DSBs) in their DNA, which are difficult for cells to repair. When these DSBs accumulate, they trigger cell death pathways such as apoptosis, mitotic catastrophe, or senescence [2,3].

Radiotherapy, often metaphorically described as a ’sniper’ in cancer treatment, mirrors the precision and accuracy of a sniper’s perfect shot. This comparison underscores the meticulous nature of radiotherapy research, which is dedicated to increasing the rate of cancer cell death during treatment while minimising damage to healthy tissues. This research focuses on improving the precision of radiation beams and delivering the right dose to the right location every time [4].

Radiotherapy research is a dynamic field constantly developing and improving therapeutic options. These improvements span various aspects, from delivering treatment [5,6] and enhancing imaging and tracking [7,8] to improving targeting precision [9]. The collective effect of these advancements is broadening the radiation therapeutic window, which minimises harm to healthy tissues. However, the role of radiotherapy extends beyond the ’sniper’ metaphor [10,11,12]. While the classical modes of cell death are central to the therapeutic effects of radiation treatment, they do not fully explain the long-term effects of radiation on tumours or the resistance observed in some cancers. Recent advances in cancer biology have expanded our understanding of radiotherapy beyond direct DNA damage, revealing its significant impact on tumour metabolism and autophagy. These processes are critical in determining how cancer cells respond to radiation, influencing survival and treatment resistance. Innovations such as FLASH radiotherapy [13,14] exploit ultra-high dose rates to minimise damage to healthy tissues while targeting cancer’s metabolic vulnerabilities. Simultaneously, nanoparticle-enhanced radiotherapy [15,16] improves precision by leveraging nanomaterials to amplify radiation effects and induce targeted DNA damage. By integrating these biological insights, radiotherapy could be transformed into a more powerful systemic treatment that leverages cancer’s metabolic vulnerability.

## 2. Radiation-Induced Metabolic Reprogramming of Glucides

One of the hallmark features of cancer metabolism is the reliance on aerobic glycolysis, where cancer cells preferentially convert glucose to lactate, even in the presence of oxygen. This aerobic glycolysis is an inefficient means of generating ATP compared to the amount obtained by mitochondrial respiration [17]. However, this pathway, known as the Warburg effect [18], allows cancer cells to rapidly generate ATP and produce intermediates necessary for biosynthesis, supporting their rapid proliferation.

**Figure 1:**
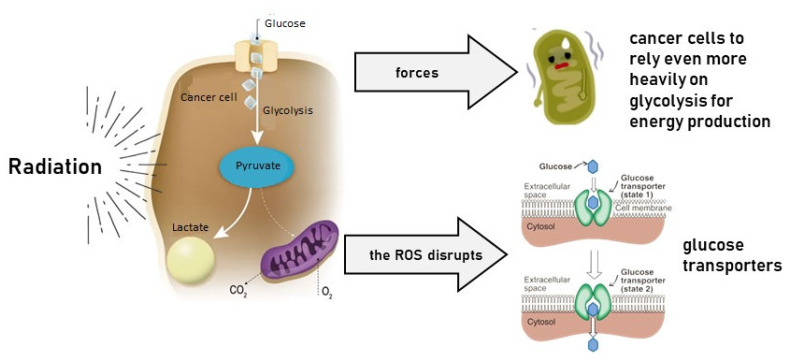
Radiation induces a metabolic vulnerability in cells due to the Warburg effect.

Radiotherapy interferes with mitochondrial function, forcing cancer cells to rely even more heavily on glycolysis for energy production [19,20]. This reliance on glycolysis makes cancer cells extremely vulnerable to disruptions in glucose metabolism (Figure 1).

At the same time, radiation, increasing the generation of reactive oxygen species (ROS), damages DNA and proteins, which can also disrupt glucose transporters essential for glucose uptake and key enzymes involved in the glycolytic pathway (hexokinase, phosphofructokinase, and pyruvate kinase).

These impairments can create a metabolic vulnerability that can be exploited with glycolytic inhibitors. These inhibitors could act synergistically with radiotherapy, amplifying the metabolic stress caused by radiation-induced mitochondrial damage.

A similar approach could be particularly effective in tumours with high glycolytic activity (such as glioblastoma, pancreatic cancer, and non-small cell lung cancer), offering a promising future for cancer treatment.

The 2-deoxy-D-glucose (2-DG), a glucose analogue, has been shown to block glycolysis by inhibiting the enzyme hexokinase, which catalyses the first step in glucose metabolism. By combining glycolysis inhibitors like 2-DG with radiation, cancer cells are deprived of their primary energy source, exacerbating the energy crisis induced by mitochondrial dysfunction and leading to increased cell death. In preclinical studies, combining 2-DG with radiation has shown promising results, increasing tumour control and reducing tumour growth compared to radiation alone.

The radiosensitising effect of 2-DG in radiotherapy has been demonstrated principally in breast, prostate, cervical, lung, and GBM cancer cells. Preclinical results have highlighted a radiosensitisation increase with 2-DG doses [21]. Dose-finding studies were undertaken to examine the tolerance and safety of the escalating 2-DG dose during the combined treatment (2-DG + RT) in glioblastoma multiforme patients. An Indian study demonstrated that oral administration of 2-DG combined with large fractions of radiation (5 Gy/fraction/week) was safe and well tolerated by glioblastoma patients without acute toxicity and late radiation damage to the normal brain, with survival benefit [22]. A single-arm multicentric phase II clinical trial showed increased survival using 2-DG combined with radiation [23]. Ongoing clinical trials are testing the safety and efficacy of this combination in various cancer types.

Other glycolysis inhibitors, such as lonidamine and gossypol, have also been investigated for their potential to enhance radiotherapy response by targeting energy metabolism. In 1994, Magno et al. reported a statistically and clinically significant increase in disease-free survival in head and neck patients treated with lonidamine and hyperfractionated radiotherapy; patients treated with combined therapy had a 2.1 times higher possibility of having a disease control at 5 years than the control group [24].

Gossypol acts as an inhibitor for several dehydrogenase enzymes, such as lactate dehydrogenase. Zerp et al. [25] showed in vitro that gossypol was a radiosensitiser on human head and neck cancer cell lines. They furthermore analysed gossypol pharmacologically in 13 head and neck cancer patients, highlighting that the plasma concentrations corresponded to those that induced radio sensitisation in vitro.

In a study by Song et al. [26], 13 patients with locally advanced oesophageal cancer who were not deemed suitable for surgery received gossypol concurrently with chemo-radiation. A complete clinical response was registered in 84.6% of patients, with an encouraging overall survival.

FDG-PET scans, using a glucose analogue to measure glucose metabolism, could detect tumour glycolytic activity and identify tumours with elevated glycolysis. They could also identify patients with better results when combined with precision radiotherapy and glycolytic inhibitors [27].

FDG-PET scans use a glucose analogue to measure glucose metabolism, detect tumour glycolytic activity, and identify tumours with elevated glycolysis. They could also be used to identify patients with better results when precision radiotherapy and glycolytic inhibitors are combined.

Certain subtypes of cancer cells (such as leukaemias, lymphomas, pancreatic ductal adenocarcinoma, high-OXPHOS subtype melanoma, and endometrial carcinoma) require an increased activity of mitochondrial oxidative phosphorylation (OXPHOS) to survive. This dependency frequently describes cancer stem cells and cells resistant to chemotherapy and targeted therapies [28]. These tumours often display increased mitochondrial activity, making them ideal candidates for treatment with mitochondrial inhibitors like metformin.

Metformin, a drug known for disrupting mitochondrial function, can significantly enhance the cytotoxic effects of radiation. Metformin can improve tumour control when combined with radiotherapy by increasing reactive oxygen species (ROS) production and reducing the tumour’s ability to generate ATP. Numerous clinical trials have explored or are currently investigating the role of metformin as an adjunct to radiotherapy in cancer treatment. A meta-analysis [29] analysed 17 studies (4 studies with prostate cancer patients, 3 with head and neck cancer, 3 with lung cancer, 3 with oesophageal tumours, 2 with rectal cancer and 1 with GBM and 1 with liver tumours) showed that diabetes patients treated with metformin and radiotherapy for tumour had a 64% higher probability of having a pathological complete response (OR = 0.36 (CI 95% 0.15–0.87), *p* = 0.02) and yielded a 62% higher probability of having a 5-year overall survival.

In cancers that often rely on oxidative phosphorylation, precision radiotherapy combined with metformin has shown promise in preclinical studies. This further highlights the role of metformin in disrupting mitochondrial function and enhancing the effects of radiotherapy.

Another metabolic feature of many tumours is the accumulation of lactate, a byproduct of glycolysis that contributes to creating an acidic microenvironment. This acidic environment promotes immune evasion and tumour invasion, making these tumours resistant to standard therapies [30].

Radiotherapy can suppress the enzymatic activity of the M2 isoform of pyruvate kinase (PKM2), which converts phosphoenolpyruvate to pyruvate [31]. Therefore, radiotherapy can disrupt the cancer’s metabolic support system by targeting lactate production or its effects on the tumour microenvironment. Drugs that inhibit lactate dehydrogenase (LDH), an enzyme that converts pyruvate to lactate, can reduce lactate accumulation and increase the tumour’s susceptibility to radiation. In preclinical studies, the LDH inhibitor galloflavin is known to enhance efficacy in radiation therapy. Koukourakis et al. assessed the effects of LDH blockade on the treatment sensitivity of glioblastoma cell lines [32]. Yang et al. found that radiotherapy increased lactate concentrations in the tumour microenvironment, leading to localised immunosuppression, and the selective LDH inhibitor, when used concurrently with radiation, improved antitumoral T-cell response and reduced tumour progression [33]. This research result suggests an intriguing relationship between metabolic pathways and immunology, avoiding the observed radiotherapy-induced immunosuppression.

Dichloroacetate (DCA), an inhibitor of pyruvate dehydrogenase kinase, is being studied for its ability to shift cancer cells away from glycolysis and increase their sensitivity to radiotherapy. Most evidence in preclinical in vitro and in vivo models shows that DCA might be beneficial in human cancer [34]. Dong et al. found that DCA radiosensitised oesophageal carcinoma cells through increased ROS accumulation in vitro and in vivo [35]. Similar radiosensitising effects were observed on gliomas and breast cancer [36]. In clinical practice, there were anecdotal positive experiences in using DCA alone or with other drugs and/or radiation [37]; however, there is unclear evidence, and ongoing clinical trials could give us further information. A phase II study [38] adding DCA to cisplatin-based CRT in locally advanced head and neck cancer compared to placebo showed that this combination was safe with no detrimental effect on survival and expected metabolite changes.

Furthermore, there was a significantly higher complete response rate in the DCA group than those treated with placebo (DCA 71.4%; *n* = 15 vs. Placebo 37.5%; *n* = 9, *p* = 0.0362). DCA could lead to enhanced reactive oxygen species (ROS) production. ROS are the primary effector molecules of radiation, and an increase hereof will enhance the radioresponse. The damage caused by ROS can initiate mitophagy, a specialised form of autophagy that selectively removes damaged mitochondria. This is a double-edged sword: on the one hand, it helps cells survive by clearing dysfunctional mitochondria, but on the other, excessive mitophagy can deplete energy reserves and lead to cell death [39].

## 3. Radiation-Induced Metabolic Reprogramming of Lipids

Lipid metabolism supports cancer cells’ rapid growth, survival, and proliferation [40]. Cancer cells reprogram their lipid metabolism to meet the increased demands for membrane synthesis, energy production, and signalling pathways essential for tumour progression. Radiotherapy significantly impacts lipid metabolism in multiple ways.

**Figure 2:**
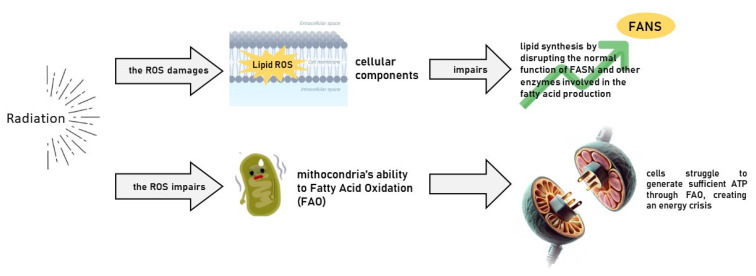
Radiation induces a metabolic vulnerability in cells, causing lipid synthesis.

Cancer cells rely heavily on de novo lipid synthesis to produce fatty acids essential for building cell membranes, producing lipid-based signalling molecules, and storing energy. The enzyme fatty acid synthase (FASN) [41] plays a central role in this process, catalysing the synthesis of fatty acids from acetyl-CoA and malonyl-CoA (Figure 2). In many cancers, FASN is overexpressed, driving tumour growth and survival. Radiation therapy causes oxidative stress, generating reactive oxygen species (ROS) that can damage cellular components, including lipids [42]. This oxidative damage impairs lipid synthesis by disrupting the normal function of FASN and other enzymes involved in fatty acid production. Furthermore, radiation-induced DNA damage and cell stress increase the energy requirements of cancer cells, making them more dependent on lipid synthesis for membrane repair and energy production.

It is demonstrated in vitro that radiation therapy reprograms the lipid metabolism of glioblastoma, generating unsaturated fatty acids. This excess promotes lipid droplet accumulation to prevent endoplasmatic reticulum stress and limit apoptosis of irradiated cells [43].

Targeting fatty acid synthesis pathways can enhance the cytotoxic effects of radiotherapy. FASN inhibitors, such as orlistat or TVB-2640, reduce the availability of lipids needed for membrane synthesis and energy storage, increasing the vulnerability of cancer cells to radiation-induced damage. Studies have shown that combining radiotherapy with FASN inhibitors increases cancer cell death [44,45,46], particularly in tumours with elevated FASN expression, such as breast, prostate, and ovarian cancers. In a recent study, TVB-2640 demonstrated a favourable tolerability profile as monotherapy and in combination with a taxane, with no significant clinical chemistry or gastrointestinal toxicities. Most Treatment Emergent Adverse Events (TEAEs) were Grade 1 or 2 in intensity, non-serious, and manageable [47].

Fatty acid oxidation (FAO) is another critical pathway in lipid metabolism, where fatty acids are broken down in the mitochondria to generate ATP, the cell’s primary energy currency [48]. Cancer cells, especially in nutrient-poor or hypoxic environments, often rely on FAO to meet their energy needs and survive under stress. Radiotherapy damages the mitochondria, impairing their ability to perform oxidative phosphorylation (OXPHOS) and FAO. As radiation increases ROS levels and disrupts mitochondrial function, cancer cells may struggle to generate sufficient ATP through FAO, creating an energy crisis that further weakens them.

Inhibitors of FAO, such as etomoxir, can block the entry of fatty acids into mitochondria, further disrupting energy production in cancer cells [49]. When combined with radiotherapy, FAO inhibition exacerbates the energy deficit in cancer cells, leading to increased cell death. This approach is particularly effective in tumours relying on FAO, such as specific prostate cancer and glioblastoma subtypes.

Radiotherapy generates ROS, which initiates lipid peroxidation in cancer cells. This oxidative damage compromises the structural integrity of the cell membrane, making it difficult for cancer cells to maintain their normal function. The accumulation of lipid peroxides can also trigger regulated cell death called ferroptosis [50], distinct from apoptosis and necrosis. The iron-dependent accumulation of lipid peroxides drives ferroptosis, and radiotherapy can catalyse this process.

To enhance radiotherapy’s lipid peroxidation effect, researchers are exploring ferroptosis inducers or ROS amplifiers, such as erastin or RSL3, combined with radiation [51]. These agents increase lipid peroxidation and push cancer cells toward ferroptosis, making them more sensitive to radiation-induced oxidative damage. This strategy holds promise for tumours resistant to traditional radiotherapy due to their ability to repair DNA damage but remain vulnerable to ferroptosis.

Lipid droplets are intracellular organelles that store neutral lipids, such as triglycerides and cholesterol esters, which can be mobilised for energy production or membrane synthesis. Cancer cells often accumulate lipid droplets as a protective mechanism to survive metabolic stress, particularly in response to treatments like radiotherapy. Cancer cells can utilise these lipid droplets as energy reserves when disrupting other metabolic pathways [51].

Radiotherapy can indirectly increase lipid droplet formation as cancer cells attempt to sequester excess fatty acids and store them in droplets to avoid ROS-induced lipid peroxidation [52].

This accumulation of lipid droplets helps cancer cells buffer the oxidative stress caused by radiotherapy and survive in hostile conditions.

Inhibiting lipid droplet formation can make cancer cells more susceptible to radiation. Triacsin C, an inhibitor of lipid droplet synthesis, prevents the storage of fatty acids in lipid droplets, increasing the likelihood of lipid peroxidation and cell death [53]. Combining radiotherapy with lipid droplet inhibitors may overcome tumour resistance mechanisms and improve treatment outcomes [54].

## 4. Radiation-Induced Metabolic Reprogramming of Amino Acids

Cancer cells reprogram amino acid metabolism to support rapid proliferation, maintain redox balance, and fuel biosynthetic pathways necessary for nucleotide, protein, and lipid synthesis [55,56]. Radiotherapy also affects amino acid metabolism, leading to changes that influence cancer cells’ response to treatment.

## 5. Radiotherapy’s Impact on Glutamine Metabolism

Glutamine is one of the most crucial amino acids for cancer cell metabolism [57]. It acts as a carbon and nitrogen source for biosynthetic processes, supports energy production, and plays a crucial role in maintaining redox balance through the synthesis of glutathione (GSH), a major antioxidant in cells.

**Figure 3:**
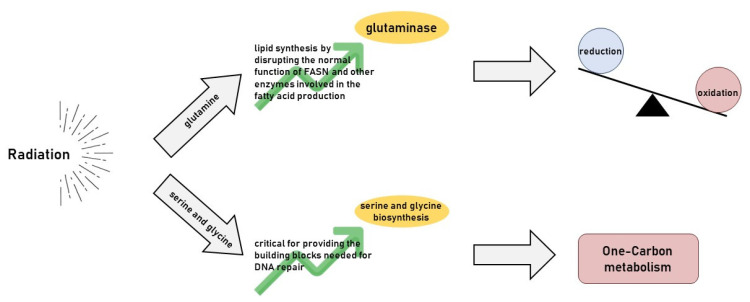
Radiation induces a metabolic vulnerability in amino acid pathways.

Radiotherapy increases the production of reactive oxygen species (ROS), leading to oxidative stress in cancer cells (Figure 3). Cancer cells rely on glutamine to maintain their redox balance by fuelling glutathione synthesis to survive this stress. The enzyme glutaminase (GLS) converts glutamine to glutamate, which is subsequently used in the synthesis of GSH, which helps detoxify ROS and repair radiation-induced damage [58].

Inhibiting glutamine metabolism disrupts the ability of cancer cells to produce glutathione, making them more vulnerable to radiation-induced oxidative damage. Glutaminase inhibitors, such as CB-839, block the conversion of glutamine to glutamate, depriving cancer cells of the necessary precursors for GSH production [55]. As a result, the cancer cells’ ability to neutralise ROS is compromised, leading to increased oxidative stress and enhanced cell death following radiotherapy. This approach is particularly effective in glutamine-dependent cancers, such as pancreatic and colorectal cancers.

## 6. Radiotherapy’s Impact on Serine and Glycine Metabolism

Serine and glycine are non-essential amino acids that play crucial roles in one-carbon metabolism [59]. This pathway supplies one-carbon units for nucleotide synthesis, methylation reactions, and redox homeostasis. Cancer cells often upregulate serine and glycine biosynthesis (Figure 3) to meet the high demands of nucleotide and protein synthesis required for rapid proliferation [60].

Radiotherapy induces DNA damage, increasing the demand for nucleotide synthesis as cancer cells attempt to repair the damage. Serine and glycine, through their involvement in, are critical for providing the building blocks needed for DNA repair. In addition, the serine biosynthesis pathway contributes to redox balance by generating NADPH, which is essential for maintaining GSH levels and neutralising ROS.

Targeting the enzymes involved in serine and glycine metabolism can disrupt the ability of cancer cells to repair DNA and manage oxidative stress following radiotherapy. Phosphoglycerate dehydrogenase (PHGDH), the enzyme that catalyses the first step in serine biosynthesis, is often upregulated in cancer cells [61]. Inhibiting PHGDH or other enzymes in the serine–glycine pathway reduces the availability of one-carbon units required for nucleotide synthesis, impairing DNA repair and increasing the sensitivity of cancer cells to radiotherapy. This approach has shown promise in cancers that heavily rely on serine biosynthesis, such as breast cancer and melanoma.

## 7. Radiotherapy’s Effect on Arginine Metabolism

Arginine is a semi-essential amino acid involved in several key metabolic pathways, including the synthesis of nitric oxide (NO) and polyamines [62]. These play roles in cell proliferation, immune modulation, and tumour progression. Arginine metabolism is often altered in the microenvironment to support tumour growth and suppress anti-tumour immune response [63].

Radiotherapy can influence arginine metabolism by modulating the tumour microenvironment and impacting immune cell function [64]. Tumour-associated macrophages (TAMs) and myeloid-derived suppressor cells (MDSCs) metabolise arginine to produce NO, promoting tumour growth and creating an immunosuppressive environment [65,66]. Additionally, MDSCs deplete arginine from the microenvironment, limiting the availability of this amino acid to immune cells such as T-cells, thereby reducing their ability to mount an effective anti-tumour response.

Depriving cancer cells of arginine can disrupt tumour metabolism and reverse immune suppression [67]. Arginine-depleting enzymes, such as arginine deiminase (ADI), degrade arginine and can potentially limit tumour growth [68]. When combined with radiotherapy, arginine depletion impairs tumour cell proliferation and enhances the anti-tumour immune response by reducing immunosuppression [69,70]. This combination approach can be particularly effective in cancers resistant to radiotherapy due to immune evasion, such as melanoma and hepatocellular carcinoma.

## 8. Radiotherapy’s Interaction with Asparagine Metabolism

Asparagine is another non-essential amino acid that plays a role in cancer cell survival, particularly in regulating protein synthesis under nutrient-stress conditions [71]. Some cancer cells, especially those in nutrient-poor environments, rely on asparagine to sustain protein synthesis and survival.

Radiotherapy creates metabolic stress by increasing the demand for amino acids to repair radiation-induced damage. Asparagine is required for protein synthesis and cellular stress responses, and cancer cells that upregulate asparagine synthetase (ASNS), the enzyme that synthesises asparagine, may better withstand the effects of radiotherapy [72].

Asparaginase, an enzyme that breaks down asparagine, has been used in treating acute lymphoblastic leukaemia (ALL) but is now being explored in solid tumours. By depleting asparagine, asparaginase limits the availability of this critical amino acid, reducing protein synthesis and increasing the sensitivity of cancer cells to radiotherapy. This approach could be particularly useful in cancers highly dependent on asparagine, such as pancreatic and ovarian cancers [73].

## 9. Radiotherapy’s Interaction with Branched-Chain Amino Acid (BCAA)

Branched-chain amino acids (BCAAs), including leucine, isoleucine, and valine, play crucial roles in protein synthesis, cell growth, and metabolism [74]. Cancer cells often increase the uptake and metabolism of BCAAs to support their anabolic needs [75]. The enzyme branched-chain amino acid transaminase 1 (BCAT1) catalyses the breakdown of BCAAs and is frequently upregulated in tumours [76]. Radiotherapy disrupts metabolic processes in cancer cells, including BCAA metabolism. Cancer cells that rely on BCAAs for growth and energy production are likely to experience metabolic stress following radiation, impairing their ability to proliferate. Inhibiting BCAT1 or other enzymes involved in BCAA metabolism can disrupt protein synthesis and energy production, increasing the susceptibility of cancer cells to radiotherapy. This approach is particularly relevant in cancers with high BCAA metabolism, such as gliomas and lung cancers.

## 10. Conclusions

Advances in metabolic profiling enable more personalised approaches to radiotherapy, allowing radiation oncologists to tailor treatment based on a tumour’s metabolic profile.

The integration of radiotherapy with an understanding of tumour metabolism represents a promising new frontier in cancer treatment. By tailoring radiotherapy to each tumour’s specific metabolic and genetic characteristics, clinicians can maximise the therapeutic effects while minimising harm to normal tissues. Developing metabolic inhibitor combinations can significantly enhance radiotherapy’s efficacy and improve patient outcomes, particularly in treatment-resistant cancers and metabolic characterisation of tumours, to tailor radiotherapy treatments to the specific biological characteristics of each patient’s cancer (Table 1). This approach will allow for more accurate targeting of tumour cells while sparing healthy tissues and significantly improving treatment outcomes. The radiation fractionation can obtain different interactions with metabolism. A conventional fractionated radiotherapy disrupts metabolic adaptations, forcing cancer cells to continuously manage energy deficits, oxidative stress, and DNA repair. Fractionated doses can be particularly effective in cancers that rely heavily on glycolysis or fatty acid metabolism, as repeated radiation exposure depletes their energy reserves and overwhelms their metabolic capacity. This is particularly relevant in highly glycolytic tumours, such as glioblastomas and pancreatic cancers, where fractionated radiation progressively depletes cellular ATP stores.

Larger doses of radiation, instead, cause immediate and severe mitochondrial dysfunction, leading to a rapid depletion of ATP and increased ROS production. This acute metabolic stress overwhelms cancer cells’ capacity to maintain energy homeostasis and exacerbates oxidative damage.

Hypofractionation disrupts multiple metabolic pathways at once, such as glycolysis, lipid metabolism, and amino acid metabolism. When these pathways are impaired by radiation, cancer cells face an acute energy crisis. This leads to metabolic bottlenecks that cancer cells cannot bypass in the short recovery time between larger radiation doses.

A high dose of radiation delivered in Sterotactic Body Radiation Therapy (SBRT) rapidly disrupts cancer cells’ metabolic pathways, leading to immediate mitochondrial damage, a sharp increase in ROS, and widespread ATP depletion. The severe oxidative stress can trigger ferroptosis, a form of cell death caused by lipid peroxidation, which is highly dependent on cellular metabolism. SBRT forces cancer cells to metabolically collapse, as the high radiation dose overwhelms their ability to reprogram metabolism to cope with the damage. Cancer cells that rely on glutamine, glucose, or fatty acids for survival are particularly vulnerable, as SBRT severely limits their ability to use these resources for energy and redox maintenance.

Metabolic imaging techniques, such as PET scans using fluorodeoxyglucose (FDG) or amino acid tracers, can provide real-time insights into how a tumour’s metabolism shifts in response to treatment. Tumours that show persistent metabolic activity (e.g., continued glucose uptake or amino acid metabolism) may require higher radiation doses or metabolic inhibitors to prevent treatment resistance. Adaptive radiotherapy can target tumours that develop metabolic resistance during treatment. For example, tumours that switch from glycolysis to fatty acid oxidation in response to radiation can be targeted with FAO inhibitors or lipid metabolism blockers. This dynamic approach allows clinicians to fine-tune the treatment plan based on the tumour’s evolving metabolic landscape.

Advancements in understanding the metabolic-modulating effects of radiotherapy open up new avenues for enhancing treatment efficacy and personalising approaches for diverse cancer types. The future of radiotherapy lies in its integration with emerging technologies and refined biological insights:

Metabolic Imaging for Personalised Radiotherapy: real-time imaging technologies will enable precise mapping of tumour metabolism to identify metabolic vulnerabilities and guide the use of metabolic inhibitors alongside radiotherapy for maximum tumour response.

Integration with Systemic Therapies: combining radiotherapy with metabolic inhibitors, immunotherapy, and nanotechnology-based drug delivery systems can synergize effects.

Exploitation of Unique Tumour Metabolic Pathways: targeting cancer-specific metabolic dependencies, such as glycolysis, fatty acid oxidation, or amino acid metabolism, will enhance radiosensitisation.

Exploration of FLASH Radiotherapy: the ultra-high dose rate of FLASH radiotherapy represents a groundbreaking approach to reduce damage to healthy tissues while effectively targeting tumours.

Immune Modulation and Ferroptosis Induction: emerging research on radiotherapy-induced ferroptosis and immunomodulation highlights the potential for exploiting oxidative stress and lipid peroxidation to overcome treatment resistance.

By leveraging these innovations, radiotherapy has the potential to transition from a primarily localised treatment to a systemic approach that addresses cancer’s complex metabolic and immunological landscape.

## Figures and Tables

**Table 1:** Overview of metabolic pathways affected by radiotherapy.

Metabolic Pathway	Function in Cancer	Effect of Radiotherapy	Inhibitors
Glycolysis	Rapid energy production via glucose conversion to lactate (Warburg effect)	Increased reliance on glycolysis due to mitochondrial dysfunction	2-DG, Lonidamine, Gossypol
Oxidative Phosphorylation (OXPHOS)	Energy production through mitochondrial activity	Mitochondrial damage impairs ATP generation	Metformin
Fatty Acid Synthesis	Lipid production for membranes and energy storage	Disruption of lipid synthesis pathways	Orlistat, TVB-2640
Fatty Acid Oxidation (FAO)	Breakdown of fatty acids for energy, especially in hypoxic conditions	Impaired FAO due to mitochondrial damage	Etomoxir
Lactate Metabolism	Lactate accumulation supports tumour microenvironment, immune evasion	Increased acidity in the tumour microenvironment	Galloflavin, DCA
Glutamine Metabolism	Supports nucleotide synthesis, redox balance (glutathione production)	Increased oxidative stress depletes glutathione	CB-839 (Glutaminase inhibitor)
Serine and Glycine Metabolism	One-carbon metabolism for nucleotide synthesis, redox balance	Increased demand for nucleotide synthesis for DNA repair	PHGDH inhibitors
Arginine Metabolism	Polyamine and nitric oxide production, immune modulation	Modifies immune responses and NO production	Arginine Deiminase (ADI)
